# Bayesian regularization to predict neuropsychiatric adverse events in smoking cessation with pharmacotherapy

**DOI:** 10.1186/s12874-023-01931-7

**Published:** 2023-04-29

**Authors:** Van Thi Thanh Truong, Charles Green, Claudia Pedroza, Lu-Yu Hwang, Suja S. Rajan, Robert Suchting, Paul Cinciripini, Rachel F. Tyndale, Caryn Lerman

**Affiliations:** 1 Department of Cardiothoracic and Vascular Surgery, McGovern Medical School, The University of Texas Health Science Center at Houston, 6400 Fannin Street, Suite 2850, Houston, TX 77030 USA; 2 Center for Clinical Research and Evidence-Based Medicine, Department of Pediatrics, McGovern Medical School, The University of Texas Health Science Center at Houston, Houston, TX USA; 3 Department of Epidemiology, Human Genetics and Environmental Sciences, School of Public Health, The University of Texas Health Science Center at Houston, Houston, TX USA; 4 Department of Management, Policy and Community Health, School of Public Health, The University of Texas Health Science Center at Houston, Houston, TX USA; 5 Faillace Department of Psychiatry and Behavioral Sciences, McGovern Medical School, The University of Texas Health Science Center at Houston, Houston, TX USA; 6 Department of Behavioral Science, The University of Texas MD Anderson Cancer Center, Houston, TX USA; 7 Centre for Addiction and Mental Health, Toronto, ON Canada; 8 Department of Pharmacology and Toxicology, University of Toronto, Toronto, ON Canada; 9 Keck School of Medicine, USC Norris Comprehensive Cancer Center, University of Southern California, Los Angeles, CA USA

**Keywords:** Bayesian regularization, Prediction model, Model selection, Neuropsychiatric adverse events, Smoking cessation pharmacotherapy, Sleep disturbance

## Abstract

**Background:**

Research on risk factors for neuropsychiatric adverse events (NAEs) in smoking cessation with pharmacotherapy is scarce. We aimed to identify predictors and develop a prediction model for risk of NAEs in smoking cessation with medications using Bayesian regularization.

**Methods:**

Bayesian regularization was implemented by applying two shrinkage priors, Horseshoe and Laplace, to generalized linear mixed models on data from 1203 patients treated with nicotine patch, varenicline or placebo. Two predictor models were considered to separate summary scores and item scores in the psychosocial instruments. The summary score model had 19 predictors or 26 dummy variables and the item score model 51 predictors or 58 dummy variables. A total of 18 models were investigated.

**Results:**

An item score model with Horseshoe prior and 7 degrees of freedom was selected as the final model upon model comparison and assessment. At baseline, smokers reporting more abnormal dreams or nightmares had 16% greater odds of experiencing NAEs during treatment (regularized odds ratio (rOR) = 1.16, 95% credible interval (CrI) = 0.95 – 1.56, posterior probability P(rOR > 1) = 0.90) while those with more severe sleep problems had 9% greater odds (rOR = 1.09, 95% CrI = 0.95 – 1.37, P(rOR > 1) = 0.85). The prouder a person felt one week before baseline resulted in 13% smaller odds of having NAEs (rOR = 0.87, 95% CrI = 0.71 – 1.02, P(rOR < 1) = 0.94). Odds of NAEs were comparable across treatment groups. The final model did not perform well in the test set.

**Conclusions:**

Worse sleep-related symptoms reported at baseline resulted in 85%—90% probability of being more likely to experience NAEs during smoking cessation with pharmacotherapy. Treatment for sleep disturbance should be incorporated in smoking cessation program for smokers with sleep disturbance at baseline. Bayesian regularization with Horseshoe prior permits including more predictors in a regression model when there is a low number of events per variable.

**Supplementary Information:**

The online version contains supplementary material available at 10.1186/s12874-023-01931-7.

## Background

Despite sizable evidence showing that pharmacological cessation medications do not elevate risk of neuropsychiatric adverse events (NAEs), the post-marketing reports of NAEs, including those that gave rise to the warnings from the United States Food and Drug Administration (FDA) for varenicline cannot be ignored since such serious adverse events can negatively affect the cessation attempt [1–9]. Identifying risk factors and developing a predictive model for NAEs experienced when using medications for smoking cessation could be a strategy to enhance treatment adherence, improve the likelihood of quitting, and prevent relapse.

There is a scarcity of literature on risk factors for NAEs in smoking cessation with pharmacotherapy. To date, the Evaluating Adverse Events in a Global Smoking Cessation Study (EAGLES) is the only study that was formally designed to assess risk of NAEs among smoking cessation pharmacological agents [1]. A post-hoc secondary analysis of the EAGLES data was conducted to determine predictors for NAEs on both psychiatric and non-psychiatric cohorts using a sequential approach with stepwise regression as the final step [10]. Stepwise regression has been a popular method to identify predictors. However, this statistical technique has numerous drawbacks that have been well-established and reported [11–13]. Essentially, stepwise regression involves multiple hypothesis tests, which increases the probability of Type I errors or false positive rate. The final set of variables selected via stepwise regression is sensitive to the number and combination of variables to be tested, as well as the order of variable entry or deletion. Stepwise regression may potentially select nuisance variables and drop true predictors, resulting in overfitting models. The use of stepwise regression is even more problematic when the outcome is rare and/or the number of potential predictors is high or when the predictors are highly correlated.

In contrast, regularization is a machine learning technique that constrains or shrinks parameter estimates toward zero, allowing the inclusion of more variables while avoiding overfitting in the training dataset [14–16]. Regularizing parameter estimates also helps deal with multicollinearity which otherwise can cause erroneous identification of predictors. Therefore, the technique may render models with potentially superior out-of-sample performance. Regularization is especially well-suited for sparse solutions where parameter estimates are mostly zero (or very small) and only a few are indeed nonzero. In this study, we applied Bayesian regularization to a large clinical trial data on smoking cessation to identify baseline risk factors and to develop a prediction model for NAEs in a quit attempt aided with medication and/or counseling.

## Methods

### Original study overview

The Pharmacogenetics of Nicotine Addiction Treatment (PNAT) study is a randomized placebo-controlled clinical trial that examined the efficacy of nicotine patch versus varenicline, stratified by nicotine metabolite ratio (NMR) group [17]. The clinical trial was conducted at four centers: University of Pennsylvania, Center for Addiction and Mental Health/University of Toronto, State University of New York at Buffalo, and MD Anderson Cancer Center.

Eligible participants comprised 18 to 65-year-old smokers who reportedly smoked at least 10 cigarettes per day for at least 6 months and were recruited via advertisements for a free smoking cessation program. Smokers with history of psychiatric disorders or at risk of suicide were excluded. After a telephone screening, eligible smokers completed an in-person medical exam and psychiatric history, self-report measures of demographics and smoking history, and provided blood samples for NMR assessment. Participants were classified into slow metabolizers if NMR < 0.31 or normal metabolizers otherwise. Participants were randomized, stratified by NMR group and study center, to three treatment arms: nicotine patch, varenicline or placebo. Varenicline was initiated at pre-quit, one week before the target quit date as recommended for dose titration while nicotine patch was initiated on the target quit date. Varenicline was administered for 12 weeks while nicotine patch was delivered for 11 weeks.

Participants, study investigators and personnel were blinded to treatment assignment and NMR status. The primary endpoint was 7-day point prevalence abstinence at the end of treatment (week 11). Abstinence was attained if a participant reported no smoking (not even a puff) for at least 7 days before the telephone assessment at the end of treatment, with in-person biochemical confirmation for those reporting abstinence (CO < 8 ppm). Follow-ups were at 6 and 12 months.

### Psychosocial scales and side-effects checklist

In PNAT, nicotine dependence was determined with the Fagerström Test of Nicotine Dependence (FTND) [18, 19]. The Minnesota Nicotine Withdrawal Scale (MNWS) and Questionnaire on Smoking Urges – Brief (QSU-B) measured withdrawal symptoms and cravings, respectively [20–22]. QSU-B Factor 1 represents a strong desire and intention to smoke, with smoking perceived as rewarding while QSU-B Factor 2 demonstrates anticipation of relief from negative affect with an urgent desire to smoke [20]. The MNWS was not labelled a withdrawal scale but a behavior rating scale to avoid confusion about having withdrawal symptoms at baseline prior to cessation. The Positive and Negative Affect Schedule (PANAS) recorded positive and negative moods and feelings during the week before the time of administration [23].

The self-reported side-effect checklist identified occurrences of 29 common side effects associated with varenicline and nicotine patch during the treatment period, consistently with FDA labels. The side-effect checklists for varenicline and nicotine patch have been used in previous studies [24–27]. Participants rated the severity of each side effect during the week before the time of reporting on a four-level scale: none (no concerns at all), mild (no interference with usual daily activities), moderate (interference with some usual activities), severe (no normal activities are possible). Three psychosocial scales (MNWS, QSU-B and PANAS) and the side-effect checklist were administered at pre-quit date, target quit date, week 1, week 4, week 8 and week 11.

### Outcome definition

NAEs comprised severe-level irritability, depressed mood, sleep problems, anxiety, insomnia, abnormal dreams, disturbance in attention, fatigue, dizziness; and moderate-to-severe suicidal thoughts, agitation and hostility reported in the side-effect checklist. The binary outcome variable was coded as “Yes” if a patient reported any NAE at any time point from the target quit date to week 11; coded as “No” if they reported no NAE throughout this treatment period. The dichotomized composite NAE outcome reflected severity levels of interest of neuropsychiatric symptoms, based on FDA warnings.

### Statistical analysis

Analyses used multilevel logistic regression to predict the probability of experiencing at least one NAE (relative to none) during the treatment period. Bayesian inference was used to evaluate effects of predictors on NAE risk, given the data and regularized prior distributions. Implementing Bayesian regularization permits shrinkage of estimates towards zero, helping reduce the chance of selecting predictors that are not truly associated with outcomes but might have been picked up otherwise due to peculiarities in the training sample [28–30]. Moreover, regularized models can prevent regression coefficients from being poorly determined with unstable and high variances in the context of multicollinearity [14]. We adopted Bayesian approaches in prediction model development so that we could calculate predictive probabilities along with their credible intervals. Bayesian multilevel models also capture all available sources of uncertainty by incorporating variance associated with parameter estimates and clustering in the computation of predictive probabilities. In this analysis, study site was accounted for as random effect in all multilevel models.

Comparisons of baseline characteristics between those with and without NAEs used descriptive statistics and Bayesian unadjusted multilevel logistic regressions. All the scale scores were shown as median (first quartile – third quartile) and the remaining continuous variables as mean (standard deviation) while categorical variables as frequency (percentage).

In a psychosocial instrument, item score provides information on a specific aspect of a complex construct while summary score – a combination of some or all item scores – represents a more comprehensive measure and tends to be more statistically reliable [31]. Hence, two predictor models were evaluated: one model with only item scores and other with only summary scores of the psychosocial scales at baseline (FTND, MNWS, PANAS and QSU-B). Both models included demographic and clinical information available at baseline such as age, race, ethnicity, sex, marital status, education level, employment status, weekly number of drinks, nicotine metabolite ratio, obesity, carbon monoxide level, preference of menthol cigarette and treatment assignment. Both models also contained the interaction between NMR and treatment group to examine whether NMR modifies treatment effects on the risk of NAEs. Additionally, a binary variable representing neuropsychiatric symptoms at baseline was included in the two models. This variable was defined the same way as the outcome variable, using the information reported on the side-effect checklist at baseline.

Out of 1246 participants in PNAT, 1214 completed the side-effect checklists from pre-quit to end-of-treatment. After excluding 11 participants with any missing data at baseline, the sample size for the current analysis was 1203. Prior to modeling, we checked for zero- and near zero-variance or highly correlated predictors, linear dependencies and removed predictors as needed. All continuous predictors were standardized. The data was then randomly divided into a training set (used to fit the model) and a test set (used for out-of-sample evaluation) with a 7:3 ratio in the manner that preserved the outcome distribution in both sets after stratifying by study site and NMR group. Overall, the summary score model had 19 predictors or 26 dummy variables and the item score model had 51 predictors or 58 dummy variables. The number of events per variable ratio in the training set was 3.3 and 1.5 for the summary and item score model, respectively.

Bayesian regularization was employed by applying two shrinkage priors, namely the Horseshoe and the Laplace prior, on population-level effects [15, 16, 28–30].

The Horseshoe prior can be specified as

$${\beta }_{i}|{\lambda }_{i},\tau \sim Normal (0,{\lambda }_{i}^{2})$$

$${\lambda }_{i}|\tau \sim {C}^{+}(0,\tau )$$

$$\tau \sim {C}^{+}\left(\mathrm{0,1}\right)$$

Considering a linear model, each model parameter ${\beta }_{i}$ follows a Normal distribution with standard deviation ${\lambda }_{i}$, which has a half-Cauchy distribution with a common scale $\tau$. The scale $\tau$ also follows a standard half-Cauchy distribution. ${\lambda }_{i}$ is referred to as the local shrinkage parameter and $\tau$ the global shrinkage parameter. Intuitively, the global parameter $\tau$ imposes severe shrinkage and pulls all parameters including noise to zero, while the local parameters from a heavy-tailed Cauchy distribution allow strong predictors to escape the shrinkage.

Applying the Laplace prior is equivalent to applying least absolute shrinkage and selection operator or LASSO [14]. The Laplace prior can be specified as a compound exponential-normal distribution.

$${\beta }_{i}|\sigma ,\tau \sim Normal (0,{{\tau }^{2}\sigma }^{2})$$

$${\tau }^{2}|\alpha \sim Exponential({}^{{\alpha }^{2}}\!\left/ \!{}_{2}\right.)$$

Each predictor model was run with either of the two shrinkage priors, with a set of 1, 3, 5, 7 degree(s) of freedom. This acted as a manual grid search for the optimal shrinkage, starting at the highest level of shrinkage. Bayesian multilevel logistic models with a vague neutral prior following a Normal distribution with mean 0 and standard deviation 100 on population-level parameters served as comparisons. Priors followed a Normal distribution with mean 0 and standard deviation 100 for the intercept and a Half Student-t distribution with 4 degrees of freedom for the standard deviation of the random intercept. A total of 18 models were evaluated.

The Watanabe-Akaike (WAIC) and the leave-one-out cross-validation information criteria (LOOIC) that were calculated from the training set were used to select the final model [32]. We further dropped variables for a more parsimonious model based on posterior probabilities and not based on the posterior odds ratios because all the posterior odds ratios were small. In this analysis, posterior probability is the posterior probability that an odds ratio is larger than 1, indicating probability of harm given that the outcome is adverse.

We examined model convergence using trace plots and Rhat, and assessed the final model fit. Posterior median odds ratio with 95% credible interval and posterior probability were reported for all population-level variables in the final model. We labeled the odds ratios as *regularized odds ratios* (rOR) to emphasize the use of regularization. We considered a probability of having an effect, regardless of direction, of 0.8 to be the minimum that warrants basis for clinical decision-making. Therefore, variables with probability of harm of at least 0.8 or at most 0.2 (equivalent to probability of decreasing harm of at least 0.8) were deemed important predictors. The evaluation of the final model included calibration, discrimination, and accuracy on the test set. Calibration consisted of plotting the observed proportions of participants experiencing NAEs against the predicted probability for groups defined by deciles. The receiver operating characteristic (ROC) curve and the area under the ROC curve (AUROC) assessed discrimination performance. The Brier score – the averaged squared difference between predicted and observed values – evaluated accuracy [33].

All analyses utilized R version 4.0.2 [34]. Data preprocessing used the *caret* package [35]. Bayesian regularized multilevel logistic modeling was performed in R using Stan (version 2.21, Stan Development Team) through the *brms* package [36, 37]. The ROC curve plotting and the computation of AUROC employed the *pROC* package [38]. Calibration and accuracy assessment used the *rms* package [39].

## Results

Out of 1203 participants with complete baseline and outcome data in PNAT, 119 (10%) experienced NAEs during treatment. The most common NAE component was moderate-to-severe agitation (6.2%), then moderate-to-severe hostility (3.3%). Sleep-related NAEs included severe-level sleep problems (1.9%), insomnia (1.6%) and abnormal dreams (1%). The prevalence of the remaining NAE components was less than 1% each (see Table S1 in Additional file).

Bayesian univariate analyses suggested that smokers with high probability ($\ge$ 80%) of being more likely to suffer from NAEs during treatment were those reported at baseline more angry or irritable (1 [0, 1] vs. 0 [0, 1], P(OR > 1) > 0.99), anxious or nervous (P(OR > 1) = 0.83), depressed (P(OR > 1) = 0.83), had difficulty concentrating (P(OR > 1) = 0.98), increased appetite or weight gain (P(OR > 1) = 0.98), more restless (P(OR > 1) > 0.99), impatient (1 [0, 1] vs. 0 [0, 1], P(OR > 1) = 0.99), constipated (P(OR > 1) = 0.94), sore throat (P(OR > 1) = 0.89); more insomnia or sleep problems (0 [0, 2] vs. 0 [0, 1], P(OR > 1) > 0.99) or nightmares (0 [0, 1] vs. 0 [0, 0], P(OR > 1) > 0.99); felt more distressed (P(OR > 1) = 0.97), upset (P(OR > 1) = 0.99), guilty (P(OR > 1) = 0.98), scared (P(OR > 1) = 0.86), hostile (P(OR > 1) = 0.97), irritable (P(OR > 1) > 0.99); reported higher QSU-B Factor 1 (7 [4, 13] vs. 6 [4, 10], P(OR > 1) > 0.99), Factor 2 (20 [12, 28] vs. 17 [11, 26], P(OR > 1) = 0.96), and FTND heaviness of smoking index (P(OR > 1) = 0.95); or being Hispanic (10 (8%) vs. 53 (5%), P(OR > 1) = 0.92) (Table 1). Smokers with low probability of harm (meaning high probability of decreasing harm) felt more interested (P(OR > 1) = 0.17), enthusiastic (P(OR > 1) = 0.12), proud (P(OR > 1) < 0.01), inspired (P(OR > 1) = 0.16), attentive (P(OR > 1) = 0.16), active (P(OR > 1) = 0.04); being African American (413 (38%) vs. 39 (33%), P(OR > 1) = 0.18), had higher than high school education (755 (70%) vs. 78 (66%), P(OR > 1) = 0.18), and preferred menthol cigarette (51 (43%) vs. 518 (48%), P(OR > 1) = 0.19).

**Table 1:** Baseline characteristics between smokers experiencing NAEs versus not experiencing NAEs during smoking cessation treatment period

Variables	NAEs (*n* = 119)	No NAEs (*n* = 1084)	P(OR > 1)
Number of cigarettes per day (mean (SD))	18 (7)	18 (7)	0.42
Carbon monoxide level in ppm (mean (SD))	23 (9)	23 (10)	0.38
Obesity levels (N, %)			
Normal	35 (29)	286 (26)	Ref
Underweight	0 (0)	12 (1)	0
Overweight	41 (35)	375 (35)	0.34
Obesity	43 (36)	411 (38)	0.30
MNWS items (median [Q1, Q3])			
Angry, irritable, frustrated	1 [0, 1]	0 [0, 1]	> 0.99
Anxious, nervous	0 [0, 1]	0 [0, 1]	0.83
Depressed mood	0 [0, 0]	0 [0, 0]	0.83
Desire or craving to smoke	3 [2.5, 4]	3 [2, 4]	0.59
Difficulty concentrating	0 [0, 1]	0 [0, 1]	0.98
Increased appetite, weight gain	0 [0, 1]	0 [0, 1]	0.98
Insomnia, sleep problems	0 [0, 2]	0 [0, 1]	> 0.99
Restless	0 [0, 1]	0 [0, 1]	> 0.99
Impatient	1 [0, 1]	0 [0, 1]	0.99
Constipation	0 [0, 0]	0 [0, 0]	0.94
Cough	1 [0, 2]	1 [0, 2]	0.71
Dreaming or nightmares	0 [0, 1]	0 [0, 0]	> 0.99
Sore throat	0 [0, 0]	0 [0, 0]	0.89
PANAS positive affect items (median [Q1, Q3])			
Interested	4 [3, 4]	4 [3, 4]	0.17
Excited	3 [2, 3]	3 [2, 4]	0.31
Strong	3 [2, 4]	3 [2, 4]	0.27
Enthusiastic	3 [2, 4]	3 [2, 4]	0.12
Proud	3 [2, 4]	3 [2, 4]	< 0.01
Alert	4 [3, 4]	4 [3, 4]	0.39
Inspired	3 [2, 4]	3 [2, 4]	0.16
Determined	4 [3, 4]	4 [3, 5]	0.30
Attentive	3 [3, 4]	4 [3, 4]	0.16
Active	3 [2, 4]	3 [3, 4]	0.04
PANAS negative affect items (median [Q1, Q3])			
Distressed	1 [1, 2]	1 [1, 2]	0.97
Upset	1 [1, 2]	1 [1, 2]	0.99
Guilty	1 [1]	1 [1]	0.98
Scared	1 [1]	1 [1]	0.86
Hostile	1 [1]	1 [1]	0.97
Irritable	1 [1, 2]	1 [1, 2]	> 0.99
Ashamed	1 [1]	1 [1]	0.62
Nervous	1 [1, 2]	1 [1, 2]	0.74
Jittery	1 [1]	1 [1]	0.79
Afraid	1 [1]	1 [1]	0.71
QSU-B Factor 2 (median [Q1, Q3])	7 [4, 13]	6 [4, 10]	> 0.99
QSU-B Factor 1 (median [Q1, Q3])	20 [12, 28]	17 [11, 26]	0.96
Experience neuropsychiatric events (N, %)	2 (1.7)	24 (2.2)	0.29
Age (mean (SD))	46 (12)	46 (11)	0.64
Race (N, %)			
Caucasian	69 (58)	596 (55)	Ref
African American	39 (33)	413 (38)	0.18
Other	11 (9)	75 (7)	0.70
Hispanic (N, %)	10 (8)	53 (5)	0.92
Female (N, %)	56 (47)	472 (44)	0.78
Marital status (N, %)			
Never married	42 (35)	364 (34)	Ref
Divorced/Widowed/Separated	27 (23)	283 (26)	0.23
Married or living as married	50 (42)	437 (40)	0.49
Higher than high school education (N, %)	78 (66)	755 (70)	0.18
Employment status (N, %)			
Full-time	55 (46)	496 (46)	Ref
Part-time	18 (15)	182 (17)	0.31
Retired/Unemployed	46 (39)	406 (38)	0.55
Weekly number of drinks (mean (SD))	3 (5)	3 (5)	0.50
Prefer menthol cigarette (N, %)	51 (43)	518 (48)	0.19
FTND HSI (median [Q1, Q3])	4 [3, 4]	4 [3, 4]	0.95
Treatment (N, %)			
Placebo	40 (34)	350 (32)	Ref
Varenicline	40 (34)	367 (34)	0.42
Nicotine patch	39 (33)	367 (34)	0.38
Nicotine metabolism levels (N, %)			
Slow	64 (54)	577 (53)	Ref
Normal	55 (46)	507 (47)	0.45

*NAEs* Neuropsychiatric Adverse Events, *MNWS* Minnesota Withdrawal Scale, *PANAS* Positive and Negative Affect Scale, *QSU-B* Questionnaire on Smoking Urges – Brief (Factor 1 strong desire and intention to smoke, with smoking perceived as rewarding; Factor 2 anticipation of relief from negative affect with an urgent desire to smoke), *FTND HSI* Fagerström Test of Nicotine Dependence Heaviness of Smoking Index, P(OR > 1) Posterior Probability of Being More Likely to Experience NAEs, *SD* Standard Deviation, *Q1* First Quartile, *Q3* Third Quartile, *Ref* Reference group

All models converged except for models with Laplace prior and 1 degree of freedom. The remaining models with Laplace priors needed higher number of iterations and warmups to converge. Models with the vague Normal prior had the highest WAIC and LOOIC values (see Table S2 in Additional file). Models with Horseshoe priors did better than those with Laplace priors. The item score model with Horseshoe prior and 7 degrees of freedom had the lowest values of WAIC and LOOIC. We omitted variables using three thresholds of probability less than or equal to: 0.7, 0.65 and 0.6 and checked the area under the ROC curve to select the most parsimonious model that retained the discriminative ability of the full model (see Table S3 in Additional file). We chose the model containing the variables whose posterior probability was at least 0.65, or for protective effect, whose posterior probability was at most 0.35.

The final model included five items from MNWS and two items from PANAS, as well as Factor 2 from QSU-B, after dropping variables with posterior probability within the range of 0.35 – 0.65 from the full model (Table 2). At baseline, having nightmares had an 90% probability of harm while having insomnia or sleep problems were associated with an 85% probability of harm. Smokers reporting more abnormal dreams or nightmares at baseline had 16% greater odds of experiencing NAEs during treatment (rOR = 1.16, 95% CrI = 0.95 – 1.56). Those with more severe sleep problems at baseline had 9% greater odds of experiencing NAEs (rOR = 1.09, 95% CrI = 0.95 – 1.37). Higher baseline QSU-B Factor 2 (anticipation of relief from negative affect with an urgent desire to smoke) was associated with 80% probability of harm, however, this QSU-B component incurred a very small effect (rOR = 1.01, 95% CrI = 0.99 – 1.06). Meanwhile, feeling proud has a 6% probability of harm or a 94% probability of decreasing harm, suggesting protective effect. The prouder a person felt one week before baseline resulted in 13% smaller odds of having NAEs (rOR = 0.87, 95% CrI = 0.71 – 1.02). The remaining items have posterior probability ranging from 68 to 74%, with small effects on the odds of NAEs.

**Table 2:** Regularized odds ratios and posterior probabilities of harm from the final model

Variables	Median rOR (95% CrI)	P(rOR > 1)
MNWS items		
Difficulty concentrating	1.05 (0.90 – 1.38)	0.74
Insomnia, sleep problems	1.09 (0.95 – 1.37)	0.85
Restless	1.04 (0.88 – 1.37)	0.72
Impatient	1.03 (0.88 – 1.31)	0.68
Dreaming or nightmares	1.16 (0.95 – 1.56)	0.90
PANAS items		
Proud	0.87 (0.71 – 1.02)	0.06
Irritable	1.05 (0.88 – 1.42)	0.73
QSU-B Factor 2	1.01 (0.99 – 1.06)	0.80

*MNWS* Minnesota Withdrawal Scale, *PANAS* Positive and Negative Affect Scale, *QSU-B* Factor 2 Questionnaire on Smoking Urges – Brief (anticipation of relief from negative affect with an urgent desire to smoke), *rOR* regularized Odd Ratio, *CrI* Credible Interval

**Fig. 1:**
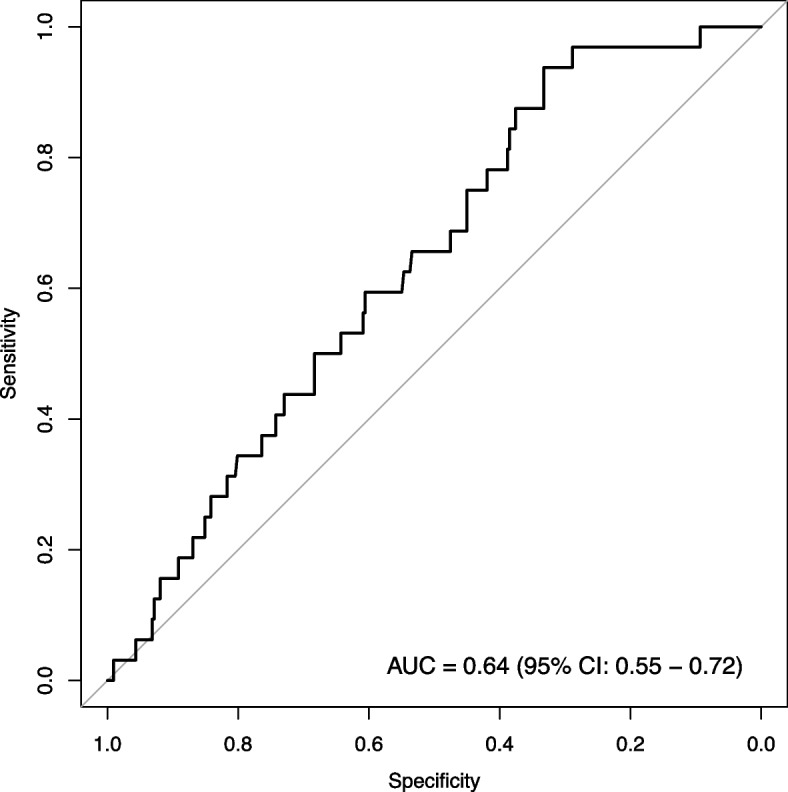
Receiver operating characteristic curve of the final model on the test set

**Fig. 2:**
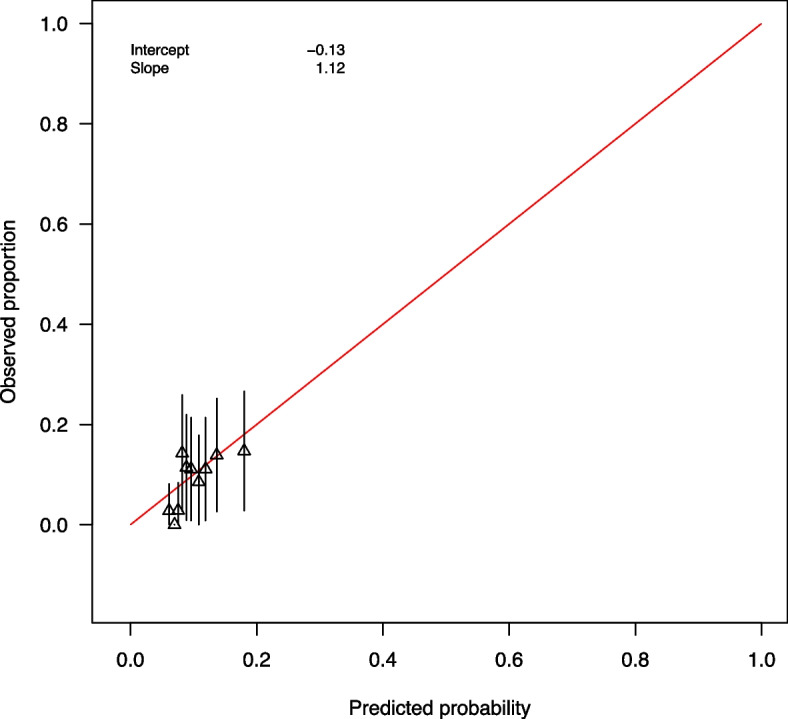
Calibration plot for the final model on the test set

Table 3 shows the final model’s performance on the test set. The final model demonstrates only poor discrimination (AUC < 0.7) in both train set (AUC = 0.66, 95% CI = 0.60 – 0.72) and test sets (Fig. 1: AUC = 0.64, 95% CI = 0.55 – 0.72). The Brier score computed on the test set is 0.08, showing that the final model is slightly more accurate than a null model (Brier score = 0.09). The calibration plot and slope indicate that the predicted probabilities derived from the model on the test set are not different enough and only spread within 0 – 0.2 (Fig. 2).

**Table 3:** Final model performance on the test set

Criteria	Value
Area under the ROC curve	0.64
Brier score	0.08
Calibration	
Intercept	-0.13
Slope	1.12

*ROC* Receiver Operating Characteristic

## Discussion

In this analysis, we attempted to build a predictive model for NAEs during smoking cessation treatment in a cohort without history of psychiatric disorders. While the final model exhibits only poor discriminative ability and most of initially considered variables either show little or no effects on the likelihood of experiencing NAEs, sleep-related symptoms at baseline were associated with probability of harm and feeling proud seems to be protective over NAEs.

Worse nightmares at baseline incurred an 90% probability of harm while more severe insomnia or sleep problems 85%. Smokers reported more abnormal dreams or nightmares at baseline had 16% greater odds of suffering from NAEs during treatment. Those with more serious sleep problems had 9% greater odds of having NAEs. One can argue that this is because there are sleep components in NAEs. While that is true, the prevalence of sleep-related side effects is small (severe sleep problems (1.9%), severe insomnia (1.6%) and severe abnormal dreams (1%)), compared to the most common NAE components which are moderate-to-severe agitation (6.2%), and moderate-to-severe hostility (3.3%).

Sleep disturbances have been observed and well-reported in smoking and smoking cessation [40]. In a population-based, nationally representative sample, current smoking status was associated with trouble falling asleep, waking up during the night and waking up too early in the morning [41]. Current smokers took more time to drift off while sleeping less than never smokers. During cessation, sleep problems can be magnified due to withdrawal symptoms and treatment side effects [42]. Sleep-related adverse events including insomnia and abnormal dreams have been well noted among varenicline users [3, 6]. Smokers using nicotine patch also reported sleep disturbance in randomized controlled trials [43]. It is established that sleep troubles can undermine quitting effort therefore decrease quit rate and/or lead to relapse. In our analysis, having more sleep-related symptoms prior to cessation resulted in 85 – 90% probability of being more likely to experience NAEs during treatment. Sleep and affect (comprising emotions, moods, stress, anxiety) have a complex symbiotic and multidimensional relationship and treating insomnia could ease symptoms in mental health disorders [44]. Sleep disturbance could be a risk factor for most mental health conditions therefore treating sleep problems is likely to be beneficial [45].

Treatment for sleep disorders should be incorporated in a smoking cessation program for smokers with sleep disorders. Cognitive behavior therapy for insomnia (CBT-I) has been proposed and investigated as a treatment for insomnia in mental health context [40, 46]. A randomized controlled trial found that CBT-I improved insomnia, which led to the reduction in paranoid and hallucinatory experiences in a large sample of college students [46]. In a randomized pilot study examining the effect of integrating CBT-I into smoking cessation counseling among 19 treatment-seeking smokers with sleep problems, despite no significant effects, the CBT-I group benefited from improvements in sleep efficiency, sleep duration, total sleep disturbance, severity of insomnia symptoms, and stayed abstinent longer than the counseling only group [47]. These results have limited interpretation due to small sample size, which warrants future studies with adequate power to detect the effect of CBT-I on sleep disturbances and NAEs during cessation, as well as smoking cessation outcomes.

We noted from our results that the greater extent to which a person had felt proud one week before baseline, the less likely that person would suffer from NAEs during cessation treatment. While there is no research specifically on feeling proud and neuropsychiatric disorders, positive affect and psychological strengths have been shown to be protective factors for mental health. Positive emotionality was negatively correlated with depressive and some panic disorders and phobias symptoms [48]. In a longitudinal cohort of active duty Army soldiers, psychological strengths or resilience, made up by seven constructs (optimism, problem-focused coping, adaptability and flexibility, positive affect, catastrophic thinking, loneliness, spirituality and meaning), were associated with reduced likelihood of being diagnosed with a psychiatric disorder in a dose–response manner [49]. Positive affect also was protective against sleep problems and suicidal ideation in older adults [50, 51].

Consistent with EAGLES and other studies, varenicline (rOR = 1.00, 95% CrI = 0.92 – 1.17, P(rOR > 1) = 0.56) and nicotine patch (rOR = 1.00, 95% CrI = 0.86 – 1.09, P(rOR > 1) = 0.45) did not show any effect on the risk of NAEs therefore were dropped in the final model [1–6]. We did not find that being White or being anxious at baseline were predictive of NAEs among non-psychiatric smokers like in EAGLES. An important difference between the two analyses lies in the outcome definition. Since EAGLES was specifically designed to evaluate NAEs in smoking cessation with pharmacotherapy, NAEs were pre-specified and prospectively collected. Meanwhile, although the side-effect checklist in PNAT is rather extensive and the manner in which a side effect was rated was similar to that in EAGLES, we could only rely on the availability of side effects collected for our definition.

We conceived and executed this secondary analysis, hoping to develop a risk prediction model for NAEs. Nonetheless, the model exhibits poor performance on both train and test sets. One reason is due to a limitation as a secondary analysis, we could not assess variables that were not collected but might be potential predictors of neuropsychiatric risk. However, as noted above, research on risk factors for NAEs in smoking cessation treatment is scarce, probably because severe NAEs are rare hence the lack of focus or interest in the topic, particularly in non-psychiatric cohort. In addition, most of the available predictors that we considered initially had very small effects on NAE risk. The small magnitude of the reported odds ratio was partially attributable to the implementation of regularization, but most posterior probabilities were close to 0.5, indicating no or little effect. Psychometric measures were self-reported therefore subject to reporting and recall bias. Biomarkers may possess greater predictive power than behavioral measures when it comes to neuropsychiatric symptoms. Still, it would be challenging to identify such biomarkers and would be even harder or impossible to apply in certain primary care settings or smoking cessation clinics. The findings in this analysis can only be applied to individuals without major psychiatric and medical comorbidities per the original study’s eligibility criteria. They also might not be generalizable to casual nondaily cigarette smokers, or non-cigarette or e-cigarette smokers.

Bayesian regularization does not automatically offer variable selection. The present analysis adapted “hard shrinkage” [15], and eliminated variables by putting a threshold on posterior probability instead of coefficient because all the posterior median of coefficients were small. The area under the ROC curve was computed for models created by a range of threshold values, and then compared to select a reasonable threshold and determine a final model that was parsimonious without losing the discriminative ability of the full model. The implementation of Bayesian regularization could be computationally expensive, time consuming and technically demanding. Fortunately, Stan and R packages including *brms* and *rstanarm* allow efficient and convenient Bayesian modeling and inference, especially for high-dimensional models [36, 37, 52]. This analysis did not explore non-linearity and any interaction other than one between NMR and treatment group.

Our original study is one of the biggest multicenter trials evaluating nicotine dependence treatment. The psychosocial scales used in this study are easily obtained, well-validated and widely used. The side effects were extensively and carefully collected. Our findings about the effect of sleep disturbances on NAEs risk emphasize the need for specialized treatment in smokers with sleep problems. The implementation of Bayesian regularization, in particular Horseshoe prior, allows inclusion of more predictors without overfitting, especially in a context of low ratio of events per predictor. Moreover, a Bayesian framework naturally incorporates all sources of uncertainty, including study site variability.

## Conclusion

We attempted to develop a predictive model for neuropsychiatric adverse events during smoking cessation treatment using Bayesian regularization. Bayesian regularization with Horseshoe prior permits including more predictors in a regression model when there is a low number of events per variable. While the model does not perform as expected, we found that more severe sleep-related symptoms reported at baseline resulted in at least 85% probability of being more likely to experience neuropsychiatric adverse events. Treatment for sleep disturbance such as cognitive behavioral therapy for insomnia should be incorporated in smoking cessation program for smokers with sleep disturbance at baseline. In contrast, feeling proud, as part of positive affect, during the week prior to cessation seemed to be protective over neuropsychiatric adverse events during treatment.

## Supplementary Information

**Additional file 1.** Supplementary result tables.

## Data Availability

Requests for the deidentified data from the Pharmacogenetics of Nicotine Addiction Treatment study can be considered by contacting the study principal investigators (CL and RFT). Analysis code can be obtained upon request made with the corresponding author (VTTT).

## Abbreviations

AUROC: Area Under the Receiver Operating Characteristic curve
CBT-I: Cognitive Behavior Therapy for Insomnia
CrI: Credible Interval
EAGLES: The Evaluating Adverse Events in a Global Smoking Cessation Study
FTND: Fagerström Test of Nicotine Dependence
LOOIC: Leave-One-Out cross-validation Information Criteria
MNWS: Minnesota Nicotine Withdrawal Scale
NAEs: Neuropsychiatric Adverse Events
NMR: Nicotine Metabolite Ratio
OR: Odds Ratio
PANAS: Positive and Negative Affect Schedule
PNAT: The Pharmacogenetics of Nicotine Addiction Treatment study
QSU-B: Questionnaire on Smoking Urges – Brief
ROC: Receiver Operating Characteristic curve
rOR: Regularized Odds Ratios
WAIC: Watanabe-Akaike Information Criteria
