# Supplementary material for: Bayesian regularization to predict neuropsychiatric adverse events in smoking cessation with pharmacotherapy
Source: BMC Med Res Methodol. 2023 Apr 29;23:107. doi: 10.1186/s12874-023-01931-7 (PMC10148544; doi:10.1186/s12874-023-01931-7)
Supplement: Supplementary file 1 — Additional file 1. Supplementary result tables. [file 12874_2023_1931_MOESM1_ESM.docx]

## SUPPLEMENTARY INFORMATION

## Bayesian Regularization to Predict Neuropsychiatric Adverse Events in Smoking Cessation with Pharmacotherapy

Van Thi Thanh Truong PhD^1^, Charles Green PhD^2^, Claudia Pedroza PhD^2^, Lu-Yu Hwang MD^3^, Suja S. Rajan PhD^4^, Robert Suchting PhD^5^, Paul Cinciripini PhD^6^, Rachel F. Tyndale PhD^7,8^, Caryn Lerman PhD^9^

1. Department of Cardiothoracic and Vascular Surgery, McGovern Medical School, Houston, Texas, USA
2. Center for Clinical Research and Evidence-based Medicine, Department of Pediatrics, McGovern Medical School, Houston, Texas, USA
3. Department of Epidemiology, Human Genetics and Environmental Sciences, University of Texas School of Public Health, Houston, Texas, USA
4. Department of Management, Policy and Community Health, University of Texas School of Public Health, Houston, Texas, USA
5. Faillace Department of Psychiatry and Behavioral Sciences, McGovern Medical School, Houston, Texas, USA
6. Department of Behavioral Science, The University of Texas MD Anderson Cancer Center, Houston, Texas, USA
7. Centre for Addiction and Mental Health, Toronto, Ontario, Canada
8. Department of Pharmacology and Toxicology, University of Toronto, Toronto, Ontario, Canada
9. USC Norris Comprehensive Cancer Center, Keck School of Medicine, University of Southern California, Los Angeles, California, USA

**Corresponding author**: Van Thi Thanh Truong (Email: [van.t.truong@uth.tmc.edu](mailto:van.t.truong@uth.tmc.edu))

Table S1. Components of Neuropsychiatric Adverse Events

| Components (n = 1,214) | N (%) |
| --- | --- |
| Suicidal thoughts | 4 (0.3) |
| Agitation | 75 (6.2) |
| Hostility | 40 (3.3) |
| Irritability | 5 (0.4) |
| Depressed mood | 4 (0.3) |
| Sleep problems | 23 (1.9) |
| Anxiety | 4 (0.3) |
| Insomnia | 20 (1.6) |
| Abnormal dreams | 12 (1.0) |
| Disturbance in attention | 5 (0.4) |
| Dizziness | 3 (0.2) |
| Fatigue | 8 (0.7) |

Table S2. Model Watanabe-Akaike and Leave-One-Out Cross Validation Information Criteria

|  | Prior distribution | Number of degrees of freedom | WAIC (SE) | LOOIC (SE) |
| --- | --- | --- | --- | --- |
| Summary score models | Normal (0, 100) | | 591.5 (42.3) | 591.7 (42.3) |
|  | Horseshoe | 1 | 564.1 (38.8) | 564.1 (38.8) |
|  |  | 3 | 564.3 (38.8) | 564.4 (38.8) |
|  |  | 5 | 564.4 (38.8) | 564.4 (38.8) |
|  |  | 7 | 564.5 (38.8) | 564.5 (38.8) |
|  | Laplace | 1 | 564.1 (38.7) | 564.2 (38.7) |
|  |  | 3 | 564.8 (38.9) | 564.9 (38.9) |
|  |  | 5 | 565.5 (39.0) | 565.5 (39.0) |
|  |  | 7 | 566.5 (39.1) | 566.6 (39.2) |
| Item only models | Normal (0,100) | | 646.9 (48.1) | 648.9 (48.2) |
|  | Horseshoe | 1 | 564.2 (38.8) | 564.3 (38.8) |
|  |  | 3 | 563.8 (38.7) | 563.9 (38.7) |
|  |  | 5 | 563.8 (38.7) | 563.9 (38.7) |
|  |  | 7 | 563.7 (38.7) | 563.8 (38.7) |
|  | Laplace | 1* | - | - |
|  |  | 3 | 563.9 (38.8) | 564.0 (38.8) |
|  |  | 5 | 564.6 (38.9) | 564.7 (38.9) |
|  |  | 7 | 565.5 (39.0) | 565.6 (39.0) |

WAIC Watanabe-Akaike Information Criteria, LOOIC Leave-One-Out Cross Validation Information Criteria

*Models did not converge

Table S3. Area Under the Receiver Operating Characteristic Curves of Candidate Models

| Model |  | Train set AUC | Test set AUC |
| --- | --- | --- | --- |
| Horseshoe (df=7) item score | Full model | 0.67 (0.61 – 0.73) | 0.63 (0.54 – 0.72) |
|  | Omit 0.4<P<0.6 | 0.67 (0.61 – 0.73) | 0.63 (0.54 – 0.72) |
|  | Omit 0.35<P<0.65 | 0.66 (0.60 – 0.72) | 0.64 (0.55 – 0.72) |
|  | Omit 0.3<P<0.7 | 0.64 (0.58 – 0.71) | 0.58 (0.47 – 0.68) |

AUC Area Under the Receiver Operating Characteristic Curve, df degree(s) of freedom, P Posterior Probability
